# Comparison of various organic acids for xylo-oligosaccharide productions in terms of pKa values and combined severity

**DOI:** 10.1186/s13068-021-01919-9

**Published:** 2021-03-16

**Authors:** Rou Cao, Xinlu Liu, Jianming Guo, Yong Xu

**Affiliations:** 1 Key Laboratory of Forestry Genetics & Biotechnology (Nanjing Forestry University), Ministry of Education, Nanjing, 210037 People’s Republic of China; 2 Jiangsu Co-Innovation Center of Efficient Processing and Utilization of Forest Resources, College of Chemical Engineering, Nanjing Forestry University, Nanjing, 210037 People’s Republic of China; 3 Jiangsu Province Key Laboratory of Green Biomass-Based Fuels and Chemicals, Nanjing, 210037 People’s Republic of China; 4 College of Chemical Engineering, Nanjing Forestry University, No. 159 Longpan Road, Nanjing, 201137 People’s Republic of China

**Keywords:** Xylo-oligosaccharide, Acid hydrolysis, Combined severity, Techno-economic analysis, PKa

## Abstract

**Background:**

Methods to produce XOS have been intensively investigated, including enzymatic hydrolysis, steam explosion, and acid hydrolysis. Acid hydrolysis is currently the most widely used method to produce XOS due to its advantages of fewer processing steps, stronger raw material adaptability, higher yield, and better reproducibility. Especially, organic acids such as acetic acid, formic acid and xylonic acid work better as compared with mineral acids. However, the catalytic mechanism of different organic acids has been little studied. In this paper, four different organic acids, including formic acid, glycolic acid, lactic acid, and acetic acid were selected to compare their hydrolytic effects.

**Results:**

Using pKa values as the benchmark, the yield of xylo-oligosaccharide (XOS) increased with the increasing value of pKa. The yield of XOS was 37% when hydrolyzed by 5% acetic acid (pKa = 4.75) at 170 ℃ for 20 min. Combined severity (CS), a parameter associated with temperature and reaction time was proposed, was proposed to evaluate the hydrolysis effect. The results of CS were consistent with that of pKa values on both the yield of XOS and the inhibitor.

**Conclusion:**

The results based on pKa values and combined severity, a parameter associated with temperature and reaction time, concluded that acetic acid is a preferred catalyst. Combining the techno-economic analysis and environmental benefits, acetic acid hydrolysis process has lower factory production costs, and it is also an important metabolite and a carbon source for wastewater anaerobic biological treatment. In conclusion, production of xylo-oligosaccharides by acetic acid is an inexpensive, environment-friendly, and sustainable processing technique.

**Supplementary Information:**

The online version contains supplementary material available at 10.1186/s13068-021-01919-9.

## Background

Agricultural and forestry waste is an important biomass and renewable resource [1]. Discussions regarding the conversion and utilization of agricultural and forestry waste, such as straw, chaff, and hardwood*,* have dominated research in recent years. China is a large agricultural country, producing about 900 million tons of agricultural waste and 140 million tons of forestry waste every year. The utilization of waste is tied to the development of the country’s economy and environment. The most common and traditional methods for this include composting and incineration. Composting is simple in operation and low in cost [2], nevertheless, the accumulated wastes tend to cause pollution and harm the environment to a large extent over a period of time. Incineration is the most widely used treatment method that effectively reduces the accumulation of wastes and generates electricity to produce heat. However, the process produces a large number of harmful gases which not only cause environmental pollution, but also cause the waste of resources [3]. The generation of greater economic and environmental benefits requires the application potential of agricultural and forestry wastes to be further explored. This was demonstrated in a number of studies that used lignocellulose found in rich amounts in agricultural and forestry waste, and is a valuable resource for industrial application. Lignocellulose, the most abundant renewable resource on earth, is a complex of cellulose, hemicellulose, lignin, extractives, and inorganic components. It is well established that lignocellulose is too recalcitrant to be utilized by micro-organisms or enzymes due to the presence of lignin seals and hemicellulose sheaths, and due to the crystallinity of cellulose [4]. Consequently, lignocellulosic biomass pretreatment, a method that unfolds the structure of lignocellulose, plays a vital role in the lignocellulosic feedstock-based biorefinery. Most widely studied pretreatment methods include physical, chemical, and biological methods. After pretreatment, hemicellulose can be converted into a mixture of oligosaccharides called xylo-oligosaccharides (XOS), an extremely valuable degradation product [5]. XOS are functional oligomers of sugars with straight as well as branched chains formed by 2–10 xylose molecules through β-1,4-glycosidic bonds [6]. At present, XOS are mainly used in food, feed, medicine, and health care products. According to the XOS consumption market share survey, the global turnover of XOS was about RMB 403 million. From this, XOS used in feed accounted for 41.54% of the market, while its use in medicine and health care products accounted for 29.01% and food and beverage accounted for 27.2%. As a type of functional oligosaccharide, XOS has attracted much attention due to its unique physiological properties. It was demonstrated in a number of studies that XOS can promote the proliferation of beneficial bacteria [7], strengthen immunity [8], prevent constipation, and lower serum cholesterol [9] among other benefits. In 2018, the European Union officially approved XOS as a new food additive.

In recent years, methods to produce XOS have been intensively investigated, including enzymatic hydrolysis [10, 11], steam explosion [12], and acid hydrolysis. Acid hydrolysis is currently the most widely used method to produce XOS due to its advantages of fewer processing steps, stronger raw material adaptability, higher yield, and better reproducibility. Especially, organic acids such as acetic acid, formic acid and xylonic acid work better as compared with mineral acids. In the current work’s preliminary studies, the preparation of XOS by organic acid has resulted in promising output. Zhang et al. [13] reported a yield of 45.91% XOSs by acetic acid at pH 2.7 and at a temperature of 150 ℃. The study used 0.64 M xylonic acid to produce 44.5% XOS at 154 ℃. Zhou et al. [14] developed an integrated and green process for co-producing xylo-oligosaccharides (XOS) and gluconic acid (GA). In their study, the highest XOS yield of 39.1% obtained from the prehydrolysis was achieved with 10% acetic acid at 150 °C for 45 min. Subsequently, 88.6% conversion of cellulose was achieved in a fed-batch enzymatic hydrolysis using a solid loading of 15%. In terms of the yield of XOS, these studies achieved significant results. The effect of different organic acid catalysts in the industrial production of XOS from wheat straw was reported in this study. Using formic acid, glycolic acid, lactic acid, and acetic acid as examples, a comparative selection method for hydrolyzed organic acids is proposed in this study.

Acidity coefficient (pKa) is an important benchmark of acids in the field of biochemistry. It is an equilibrium constant which represents the ability of an acid to dissociate hydrogen ions [15]. The pKa values affect the activity, water solubility, and spectral properties of chemicals. The four organic acids used in this study had different pKa values, and the conclusion is based on the yield of XOS and by-products. For more accurate visualization analysis, combined severity (CS) a function of reaction time, pH, and temperature was established to evaluate the acid pretreatment process. The comparison using CS avoids the influence of temperature on pKa. The conclusion at this stage was also based on the conversion rate of xylan. Finally, the whole industrial production process was projected and designed. In addition, techno-economic analysis was carried out during acid hydrolysis, and the cost of producing one-ton XOS by different acids was calculated and demonstrated. The combination of these three means of comparison makes the selection of organic acids more convenient, effective, and convincing.

## Results and discussion

### The kinetics of acidolysis by various acids and degradation of wheat straw

Four low molecular weight organic acids at 0.8 mol/L concentration were analyzed in this study. To compare their hydrolytic ability, their pKa values were taken as the benchmark (Additional file 1: Table S1).

**Fig. 1:**
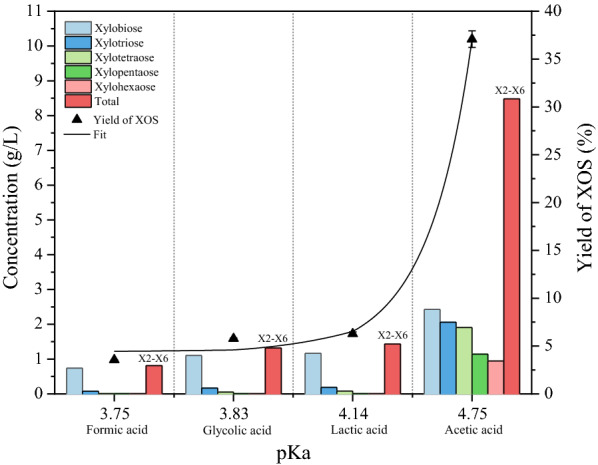
The concentration and yield of XOS with different pKa values 1

**Fig. 2:**
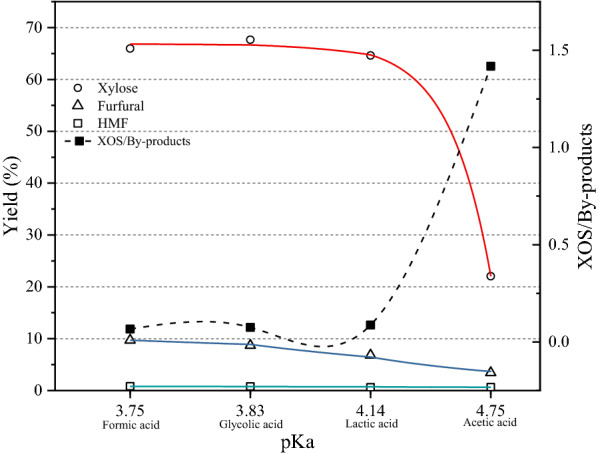
The yield of by-products with different pKa values

As observed in Fig. 1, the yield of XOSs increased with higher pKa value. When the pKa value was 4.75, the yield reached 37%. Another interesting finding was that each component of XOS (from xylo-disaccharides to xylo-hexose) increased with the increase in the value of pKa. In addition, Fig. 2 shows the yield of several by-products such as, xylose, furfural, and HMF. The yield of xylose shows a downward trend accompanied by the value of pKa ranging from 3.75 to 4.75. Notably, the ratio of XOS to the total by-products is also shown in Fig. 2. This indicates a good agreement between the pKa value and the hydrolytic efficiency of different acids.

Acidic hydrolysis of lignocellulose is a homogeneous process and can be described with the kinetic model of Arrhenius equation [16]. The Arrhenius equation is shown in Eq. (1):

1 $$k={k}^{0}\times {e}^{-\frac{\mathrm{Ea}}{RT}},$$

where *k* is kinetic constant, s^−1^; *k*^0^ is a pre-factor, s^−1^; Ea is activation energy, kJ/g mol·k; *R* is gas constant—8.314 × 10^–3^, KJ/mol K and *T* is the temperature, K.

To concretely describe the acid hydrolytic process, the Arrhenius equation was deformed using ionization constant. The equation can then be used for assessing the effect of different pKa values. The reformed equation is as followed:

2 $$y={y}^{0}\times {(\mathrm{ka})}^{m}\times {e}^{-\mathrm{Ea}/RT}.$$

In this equation, *y* represents the yield, %; *y*^0^ represents pre-factor, %; *m* is index factor; ka represents the ionization constant. The pKa value is directly related to the ionization constant as follows:

3 $$\mathrm{pKa}=-\mathrm{log}\left(\mathrm{ka}\right).$$

According to Eq. 2, the acid hydrolysis of hemicellulose is related to the ionization constant and temperature. The kinetics of hemicellulose hydrolysis at different temperatures and ka values can be determined. In this study, the temperature is kept at 170 ℃. Then use formula (3) to fit the yield of xylose in Fig. 2, and the activation energy could be calculated in Table 1. It was observed that when the hydrolysis was catalyzed by an acid with a higher pKa value, lower activation energy was required to produce xylose. The activation energy corresponds to the complexity level of the reaction. This is also supported by Figs. 1, 2 which represent that during hydrolysis with a low pKa value acid, XOS are more easily degraded to xylose, thereby reducing the yield of XOS.

**Table 1:** The activation energy value of degradation to xylose

pKa	ka (× 10^–5^)	*y*^0^ (%)	Ea (kJ/g mol k)	^a^Adjusted *R*^2^
3.75	17.78	0.52	107.12	0.973
3.83	14.79	0.76	109.21	
4.14	7.24	1.53	115.52	
4.75	1.78	5.65	131.98	

^a^Linear equation *f*(*x*) = 1.834*x* − 15.5, *R*^2^ = 0.973

**Fig. 3:**
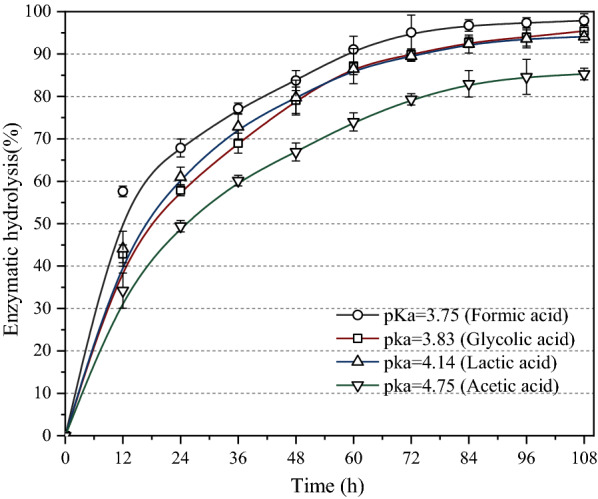
Enzymic yield of acidolysis residue with different acids

To compare the hydrolytic effects of various acids on subsequent enzymatic hydrolysis reactions, 25 FPIU/g of cellulase was used as the hydrolytic enzyme. The result obtained after 108 h of treatment is shown in Fig. 3.

When the residue was pretreated with acids of smaller pKa value and used as the substrate for enzymatic hydrolysis, the enzymatic hydrolysis rate was higher and reached 98%. However, the maximum enzymatic hydrolysis rate of the residues was 85% when hydrolyzed with acids of 4.75 pKa value, but had the highest XOS yield. On comparing the yield of XOS with enzymatic hydrolysis (Additional file 1: Figure S1), acetic acid with a pKa value of 4.75 was found to be significantly superior in terms of XOS yield as well as effectiveness. Therefore, acetic acid was the preferred catalyst for the pretreatment process.

### Establishment and application of combined severity for the evaluation of pretreatment process

The catalytic effect of acids changed with their pKa value, but the calculation function of pKa and temperature has not been determined. For more accurate visualization analysis, CS—a function of reaction time, pH, and temperature—was established to evaluate the acid pretreatment [17]. The CS is defined as follows [18]:

4 $${\text{CS}} = \log \left\{t\times{\text{exp}}\left[\left(T_{\text{H}}-{T_{\text{R}}}\right)/\upomega\right]\right\}-{\text{pH}},$$

where *t* is reaction time in min, *T*_H_ is the hydrolysis temperature in ℃, *T*_R_ is a base temperature set at 100 ℃, and *ω* is a fitting parameter which in most studies is assigned with the value of 14.75.

The pH of a weak acid decreases with an increase in temperature because of its heated ionization process, represented by the Henderson–Hasselbalch equation. Thus, a higher pH than the original value was used to calculate the CS of weak acids. In the present study, four organic acids, including formic acid, glycolic acid, lactic acid, and acetic acid were used for the pretreatment at different temperatures. The conditions and constants are shown in Table 2.

**Table 2:** Conditions and constants of four organic acids during the acidolysis

Temperature (℃)/time (min)	Acid type	pH^a^	CS
130/20	Formic acid	1.63	0.55
	Glycolic acid	1.64	0.54
	Lactic acid	1.82	0.36
	Acetic acid	2.12	0.06
130/45	Formic acid	1.51	1.03
	Glycolic acid	1.56	0.98
	Lactic acid	1.75	0.79
	Acetic acid	2.04	0.5
130/75	Formic acid	1.36	1.4
	Glycolic acid	1.41	1.35
	Lactic acid	1.54	1.22
	Acetic acid	1.89	0.87
150/20	Formic acid	1.45	0.38
	Glycolic acid	1.51	0.32
	Lactic acid	1.70	0.13
	Acetic acid	1.98	− 0.15
150/45	Formic acid	1.32	0.86
	Glycolic acid	1.42	0.76
	Lactic acid	1.58	0.6
	Acetic acid	1.87	0.31
150/75	Formic acid	1.12	1.29
	Glycolic acid	1.24	1.17
	Lactic acid	1.31	1.1
	Acetic acid	1.61	0.8
170/20	Formic acid	1.26	0.72
	Glycolic acid	1.35	0.63
	Lactic acid	1.58	0.4
	Acetic acid	1.73	0.25
170/45	Formic acid	1.12	1.20
	Glycolic acid	1.24	1.08
	Lactic acid	1.40	0.92
	Acetic acid	1.61	0.71
170/75	Formic acid	0.98	1.57
	Glycolic acid	1.11	1.44
	Lactic acid	1.17	1.38
	Acetic acid	1.45	1.10

**Fig. 4:**
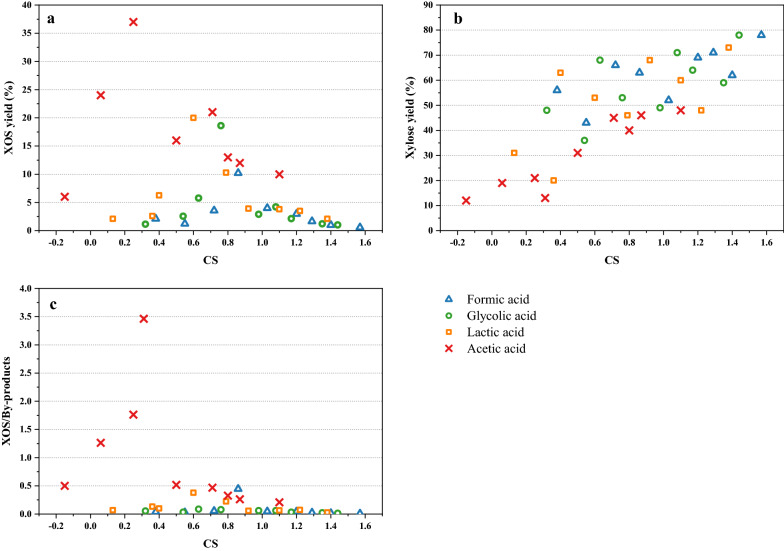
Combined severity versus XOS yield (**a**), xylose yield (**b**), XOS/by-products (**c**)

In order to evaluate the effect of pretreatment with different acids, CS was employed to describe the pretreatment condition. The value of CS has been plotted against XOS yield, xylose yield, and the ratio of XOS and by-products (Fig. 4).

The trend of XOS or xylose (by-products) yield by different acids was inconsistent and irregular, because CS is a parameter comprising temperature, pH, and time, but not acid type. Although the trend is irregular, there is no doubt that each graph shows an evident peak value. In Fig. 4a, the peak was observed with acetic acid with a value of 37%. The order of the effect of different acids on XOS yield was acetic acid > lactic acid > glycolic acid > formic acid. In contrast, the effect of acids on the peak values of xylose yield was in the opposite order—formic acid > glycolic acid > lactic acid > acetic acid. From the results of CS evaluation, it can thus be concluded that acetic acid should be preferred for either higher XOS yield or xylose (by-products) yield. Moreover, it is an excellent discovery that the results of CS evaluation are consistent with pKa values of different acids.

### Techno-economic analysis

**Fig. 5:**
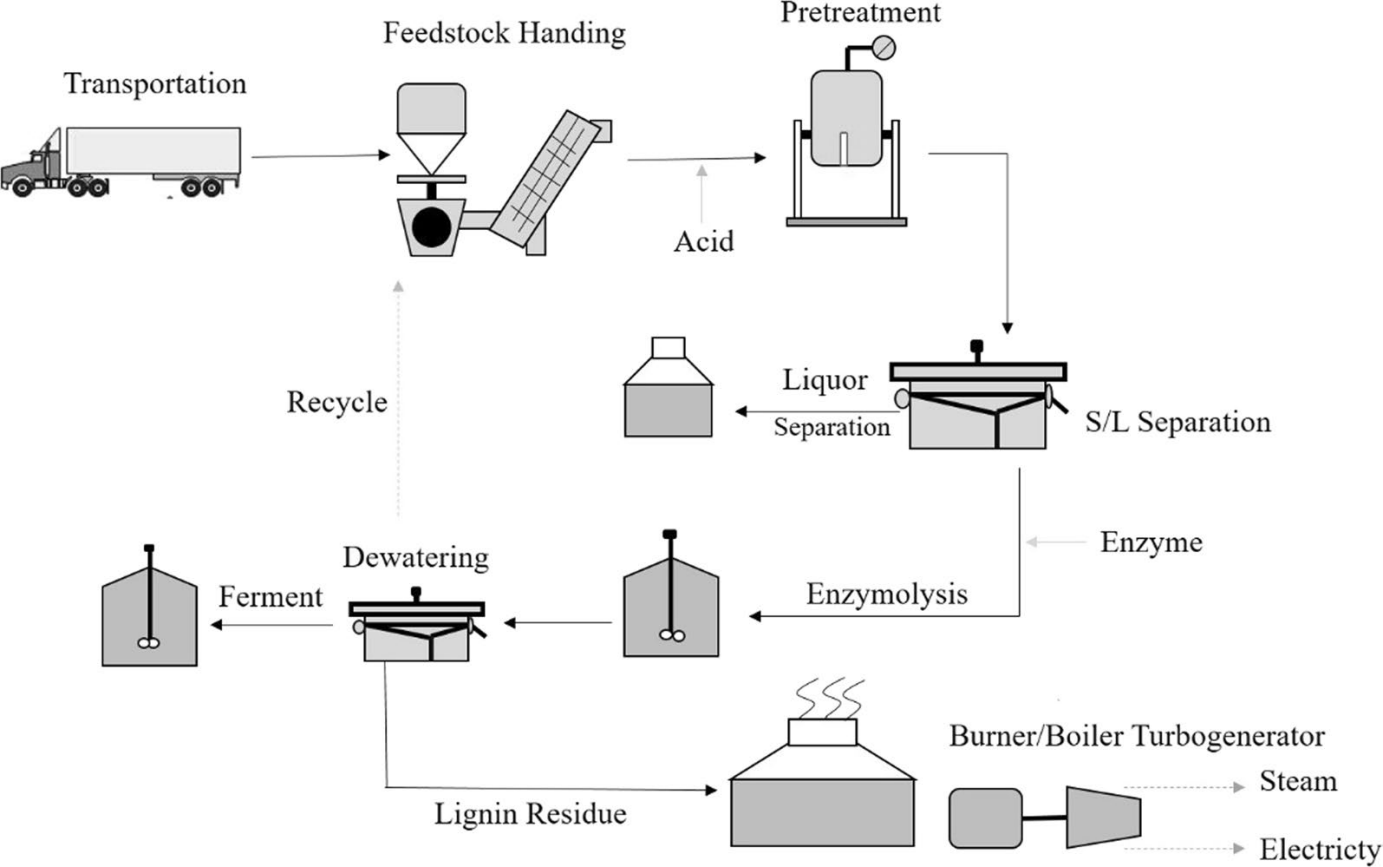
Overall industrial process

Techno-economic analysis is extremely important in research and development of lignocellulosic biofuels production processes at NREL from over two decades [19]. These include both biochemical and thermochemical approaches. In order to analyze the techno-economic aspects, the whole process of industrial application is depicted in Fig. 5.

**Fig. 6:**
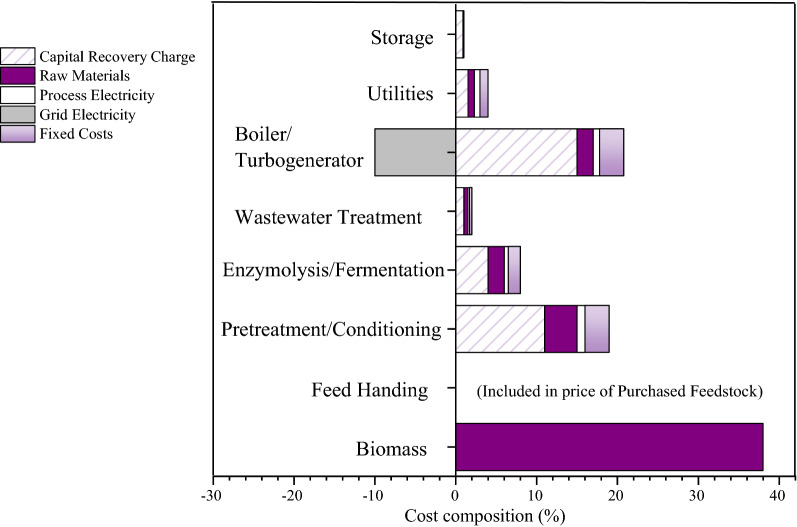
The cost composition analysis of the whole process

Baled wheat straw was delivered to the processing plant where they were broken, washed, and milled. Straw was then loaded for pretreatment at a certain (such as 1:10) solid-to-liquid ratio. After solid–liquid separation, the liquid portion was treated to obtain the target product XOS. The solid residue portion, on the other hand, goes through enzymatic degradation and fermentation process. An approximate cost composition analysis of the whole process is shown in Fig. 6.

In general, the maximum cost of the process is associated with the raw materials, which is about 38% of the production cost for one-ton XOS. It can be observed from Fig. 6 that the techno-economic difference between four different acid hydrolysis processes lies in the cost of pretreatment and conditioning, in which the ratio of capital recovery charge is 11%, raw material is 2%, process electricity is 0.8% and the fixed cost is 3%. Among these, capital recovery charge and fixed cost do not change with the type of acids. In order to compare the cost of four different acid hydrolysis processes, the price of acid is the main concern and next is the process electricity (take the best advantage of each acid in this experiment as an example). The detailed cost accounting is shown in the following table (Table 3).

**Table 3:** The detailed cost accounting

Organic acid	CRM^a^ (RMB/*t*)	CPE^b^ (RMB/*t* XOS)	Costs^c^ (RMB)
Formic acid	2800	4017.75	592.142
Glycolic acid	8000	2203.25	1617.624
Lactic acid	7800	3978.75	1591.83
Acetic acid	2260	1214.25	461.714

^a^Cost of raw material

^b^Cost of process electricity

^c^Cost = CRM × 8% + CPE × 0.2%

For each ton of XOS produced, the order of the acids in terms of the combined cost of raw materials and process electricity was as follows: glycolic acid > lactic acid > formic acid > acetic acid. Considering the production cost of the factory, acetic acid should be preferred as catalyst. Furthermore, besides the lower production costs, acetic acid has other advantages. Previous laboratory studies have shown that about 75% of acetic acid can be recovered by distillation [20]. Anaerobic biological treatment plays an important role in industrial wastewater treatment, where acetic acid [18] is a metabolite in the second stage of wastewater treatment and a carbon source in the third stage (Additional file 1: Figure S2). This has important implications in the industrial production.

## Conclusion

The hydrolytic effect of four different organic acids on wheat straw was relatively obvious. Using pKa values as the benchmark, the yield of XOS increased with the increasing value of pKa. The yield of XOS was 37% when hydrolyzed by 5% acetic acid (pKa = 4.75) at 170 ℃ for 20 min. To eliminate the impact of temperature on the dissociation constant, combined severity (CS), a parameter associated with temperature and reaction time was proposed. The results of CS were consistent with that of pKa values on both the yield of XOS and the inhibitor. In terms of techno-economic analysis, the overall cost of acetic acid was lower. For environmental benefits, acetic acid can be recovered by more than 75% and it is also an important metabolite and carbon source for wastewater anaerobic biological treatment.

## Materials and methods

### Materials

The lignocellulosic material, wheat straw, was obtained from Lianyungang city (Jiangsu Province, China). It was grouped into 20 mesh powder and was air dried to reduce the moisture under 8%. The chemical composition (wt%, based on the oven-dried weight) of the wheat straw was: 23.53% xylan, 35.34% glucan, 30.63% acid-insoluble lignin and 3.10% acid-soluble lignin.

### Acidolysis of wheat straw

This study used four different acids (formic acid, acetic acid, lactic acid, and glycolic acid) as catalysts and the concentration of each acid was 5% (w/v). The pretreatments were carried out in 35 mL stainless-steel reactors, to which 15 mL mixture, containing the substrate and the acid catalyst at a 1:10 solid-to-liquid ratio, was added. The reaction mixture was heated in a 170 ℃ oil bath for 20 min. After the reaction was complete, the supernatant was measured for the concentration of each component, including the concentration of XOS, xylose and inhibitors such as furfural. And the slag was used for enzymatic hydrolysis.

### Analytical methods

The composition of the wheat straw was determined according to the standard protocol utilized by the National Renewable Energy Laboratory (NREL). XOS (xylobiose, xylotriose, xylotetraose, xylopentaose, and xylohexaose) were analyzed using high-performance anion-exchange chromatography (HPAEC-PAD), coupled with pulsed amperometry detector (Dionex ICS-5000). The mobile phase used was 0.1 M NaOH and 0.5 M NaOAc (containing 0.1 M NaOH) at a flow rate of 0.3 mL/min and the anion-exchange column was CarboPac™ PA200.

The monosaccharide and other by-products were detected by high-performance liquid chromatography (HPLC, Agilent 1260, Agilent Technologies, Santa Clara, CA, USA) using an Aminex Bio-Rad HPX-87H column and a refractive index detector at 55 ℃, with 5 mmol/L sulfuric acid (H_2_SO_4_) as the mobile phase at a flow rate of 0.6 mL/min.

The yield of XOS was calculated according to Eq. (5):

5 $${\text{XOS yield}}\, (\%) =\frac{\text{XOS}}{\text{initial xylan content in substrate}} \times 100\%.$$

## Supplementary Information

**Additional file 1: Table S1.** The pKa values of four organic acids. **Figure S1.** The comprehensive effectiveness of XOS yield and enzymatic hydrolysis. **Figure S2.** Process of anaerobic biological treatment.

## Data Availability

All data generated and analyzed in this study are included in this published article.

## Abbreviations

XOS: Xylo-oligosaccharide
CS: Combined severity
HPLC: High-performance liquid chromatography
HPAEC: High-performance anion-exchange chromatography
NREL: National Renewable Energy Laboratory
CRM: Cost of raw material
CPE: Cost of process electricity
