# Supplementary material for: Comparison of various organic acids for xylo-oligosaccharide productions in terms of pKa values and combined severity
Source: Biotechnol Biofuels. 2021 Mar 16;14:69. doi: 10.1186/s13068-021-01919-9 (PMC7968336; doi:10.1186/s13068-021-01919-9)
Supplement: Supplementary file 1 — Additional file 1: Table S1. The pKa values of four organic acids. Figure S1. The comprehensive effectiveness of XOS yield and enzymatic hydrolysis. Figure S2. Process of anaerobic biological treatment. [file 13068_2021_1919_MOESM1_ESM.docx]

**Comparison of various orgainc acids for xylo-oligosaccharide productions in term of pKa values and combined severity**

Rou Cao ^a,b,c^, Xinlu Liu ^a,b,c^, Jianming Guo ^a,b,c^, Yong Xu ^a,b,c,*^

*^a^ Key Laboratory of Forestry Genetics & Biotechnology (Nanjing Forestry University), Ministry of Education, Nanjing 210037, People’s Republic of China*

*^b^ Jiangsu Co-Innovation Center of Efficient Processing and Utilization of Forest Resources, College of Chemical Engineering, Nanjing Forestry University, Nanjing 210037, People’s Republic of Chin*

*^c^ Jiangsu Province Key Laboratory of Green Biomass-based Fuels and Chemicals, Nanjing 210037, People’s Republic of China*

* Corresponding author at: College of Chemical Engineering, Nanjing Forestry University. No. 159 Longpan Road, Nanjing 201137, People’s Republic of China.

E-mail address: xuyong@njfu.edu.cn (Y. X.)

Tel: +86 025 85427587

Fax: +86 025 85427587

**Table S1. The pKa values of four organic acids.**

| **Organic acid** | **pKa value** |
| --- | --- |
| Formic acid | 3.75 |
| Glycolic acid | 3.83 |
| Lactic acid | 4.14 |
| Acetic acid | 4.75 |

**Fig. S1 The comprehensive effectiveness of XOS yield and enzymatic hydrolysis.**


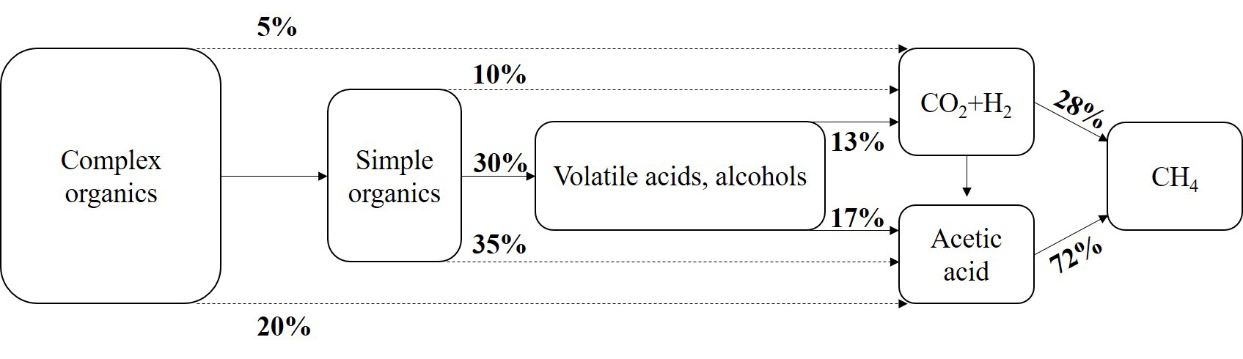


**Fig. S2 Process of Anaerobic biological treatment**
